# Cutaneous optical coherence tomography for longitudinal volumetric assessment of intradermal volumes in a mouse model

**DOI:** 10.1038/s41598-020-61276-9

**Published:** 2020-03-06

**Authors:** Kornelia Schuetzenberger, Martin Pfister, Alina Messner, Gerhard Garhöfer, Christine Hohenadl, Ulrike Pfeiffenberger, Leopold Schmetterer, René M. Werkmeister

**Affiliations:** 1 Medical University of Vienna, Center for Medical Physics and Biomedical Engineering, Währinger Gürtel 18-20, 1090 Vienna, Austria; 2 Christian Doppler Laboratory for Ocular and Dermal Effects of Thiomers, Vienna, Austria; 3 Institute of Applied Physics, Vienna University of Technology, Wiedner Hauptstr. 8-10, 1040 Vienna, Austria; 4 Medical University of Vienna, Department of Clinical Pharmacology, Währinger Gürtel 18-20, 1090 Vienna, Austria; 5 Croma Pharma GmbH, Cromazeile 2, 2100 Leobendorf, Austria; 6 Singapore Eye Research Institute, 20 College Road Discovery Tower Level 6, The Academia, Singapore, 169856 Singapore; 7 School of Chemical and Biomedical Engineering, Nanyang Technological University, 62 Nanyang Dr, Singapore, 636921 Singapore; 8 Ophthalmology and Visual Sciences Academic Clinical Program, Duke-NUS Medical School, Singapore, Singapore; 9 SERI-NTU Advanced Ocular Engineering (STANCE), Singapore, Singapore; 10 Institute of Ophthalmology, 4031 Basel, Switzerland

**Keywords:** Preclinical research, Translational research

## Abstract

Clinical evaluation of skin lesions requires precise and reproducible technologies for their qualitative and quantitative assessment. In this study, we investigate the applicability of a custom-built dermatologic OCT system for longitudinal assessment of intradermal volumes in a mouse model. The OCT, based on an akinetic swept laser working at 1310 nm was employed for visualization and quantification of intradermal deposits of three different hyaluronic acid-based hydrogel formulations – one commercial and two test substances. Hydrogels were applied in 22 BALB/c mice, and measurements were performed over a six-month time period. All hydrogels increased in volume within the first weeks and degraded steadily thereafter. The half-lifes of the test hydrogels (27.2 ± 13.6 weeks for Hydrogel 1, 31.5 ± 17.2 weeks for Hydrogel 2) were higher in comparison to the commercially available HA hydrogel (21.4 ± 12.0 weeks), although differences were not significant. The sphericity parameter was used for evaluation of the deposit geometry. While on the injection day the sphericities were similar (~0.75 ± 0.04), at later time points significant differences between the different test substances were found (T24: PRV 0.59 ± 0.09, Hydrogel 1 0.70 ± 0.11, Hydrogel 2 0.78 ± 0.07; p ≤ 0.012 for all pairs). This study shows the applicability of OCT imaging for quantitative assessment of the volumetric behavior of intradermal deposits *in vivo*.

## Introduction

In the field of dermatology, dermatoscopy and histopathology are still considered the gold standard for skin examination. While the dermatoscope provides a high-resolution view of the skin surface *in vivo*, it does not give any information about deeper layers. Histopathologic specimen, on the other hand, offer the possibility for high-resolution morphological evaluation of all layers of skin tissue but require taking tissue samples, thus being invasive. Novel imaging approaches^1^ like high-frequency ultrasound^2,3^ (HFUS), optical coherence tomography (OCT)^4,5^, photoacoustic tomography^6,7^ (PAT) and photoacoustic microscopy^8^ (PAM), reflectance confocal microscopy^9,10^ (RCM) or multiphoton microscopy^11,12^ (MPM) have gained increased attention, due to their benefits when compared to standard techniques. First and most importantly, they are non-invasive. This enables both, visualization of the unaltered tissue morphology and repetitive measurements of exactly the same structure, therefore allowing for follow-up evaluations. Due to their underlying technical principles, the methods offer different resolutions. While the superior resolution of RCM and MPM allows to resolve individual cells, their penetration depth and field of view are limited to a few hundred micrometers. PAT and PAM provide favorable penetration depth for assessment of both deeper skin tissue morphology and vasculature^13^, its resolution, however, is limited to several tenth of micrometers and measurement times for *in vivo* acquisition of three-dimensional data sets are still relatively high. OCT and HFUS, on the other hand, provide spatial resolutions ranging from a few micrometers to tens of micrometers and imaging depths of a few millimeters. Both methods also share a larger field of view of several millimeters and, despite the relatively large field of view, are very fast even when measuring larger skin sections and yield label-free cross-sectional images and volumetric datasets to a depth of several millimeters with almost cellular resolution. This results in the second major advantage of non-invasive imaging approaches: Real-time imaging producing an immediate result of the examination. However, only OCT provides this data in a non-contact manner, thus, avoiding any manipulation of the measured biological tissue. It offers both an objective and quantitative assessment of changes in skin morphology, important for follow-up evaluations in research and clinical settings and the study of therapeutic interventions.

OCT is based on interferometric principles, measuring the magnitude and echo time delay of back-reflected or back-scattered light. The penetration depth is determined by the absorption and scattering properties of the sample and depends on the measurement wavelength, while the acquired signal is given by the reflectance and scattering profile of the probed tissue^14^. As the speed of light in tissue depends on the wavelength, for absolute depth measurements, the refractive index of the tissue has to be taken into account^15,16^.

The main field of application and therefore the driver for OCT developments for skin assessment is the clinical interest for imaging skin cancer, the most common cancer worldwide. Recent research for the US estimates new incidences of 3.3 million non-melanoma skin cancers^17^ and approximately 100,000 melanoma skin cancers^18^ in 2019. For their evaluation, technologies providing a precise qualitative and quantitative assessment are needed. Although not yet applicable as a diagnostic tool, OCT provides valid information for treatment planning and monitoring. To demonstrate its usefulness and benefits compared to the current gold standard techniques, studies investigating the technical benchmarks as well as the precision and accuracy of the modality for qualitative assessment are necessary. In non-melanoma skin cancer (NMSC), epidermal thickness measurements using OCT have shown a good correlation with histology^19^ and a higher precision than HFUS assessments^20^; nevertheless both modalities overestimated the tumor thickness. In another study investigating tumor thickness in basal cell carcinoma (BCC), OCT values did not differ significantly from those measured by histopathology, while HFUS results were significantly higher^21^. De Carvalho and co-workers determined the margins of BBCs with OCT prior to Moh’s micrographic surgery and reported the benefit of OCT to better define tumor dimensions, which may help to reduce the number of surgical stages^22^.

Recently, Varkentin and co-workers performed a comparative study in humans assessing skin infiltration depths of melanocytic tissue and found a considerable agreement between histopathology and OCT or HFUS, respectively^23^. In an effort to assess the precision and accuracy of *in vivo* OCT and HFUS measurements, we recently investigated the technologies’ capabilities to visualize skin tissue morphology and to quantify predefined intradermal volumes by injecting a soft tissue filler into murine skin^24^. Soft tissue fillers, most commonly based on hyaluronic acid^25^ (HA), are naturally degraded by hyaluronidase and can serve as test substances due to their excellent biocompatibility and non-immunogenicity^26^. The longevity of such hydrogels with HA as a polymer backbone can be influenced by various factors, including their polymer concentration, viscosity or level of cross-linking^27^. In our experiments, OCT provided a higher precision in quantitative evaluation, as well as superior image quality for the assessment of tissue morphology. We therefore proposed that OCT is a suitable imaging technique for localization and quantification of volumetric changes of intradermal structures in a longitudinal manner.

The current study aimed to investigate whether dermal OCT is sensitive to detect differences in the volumetric behavior, longevity and shape of different test substances. To this end, four test substances, including a commercially available HA-filler (Princess Volume, PRV), two developmental, stabilized HA formulations (Hydrogel 1, Hydrogel 2), and a control substance (0.5% sodium-HA), were injected into the murine dermis and assessed via repeated OCT imaging over six months. In addition, on the last study day, HFUS imaging and histologic analyses were performed for confirmation of the deposit position.

## Results

Optical coherence tomography imaging of the murine skin and the injected intradermal volumes was performed in all 22 animals on all study days. The volumetric data clearly revealed the boundaries of the deposits, allowing for assessment of its volume, shape and position within the skin.

**Figure 1:**
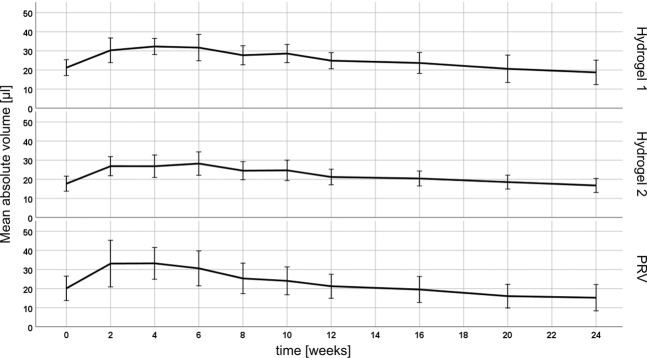
Mean absolute volume (μl) over time (weeks) of the three test substances PRV, Hydrogel 1 and Hydrogel 2 in a mouse model as measured with OCT. Error bars represent the standard deviation.

The mean initial volumes as measured with OCT, based on automatic segmentation using a convolutional neural network^28^, were 21.2 ± 4.2 μl for Hydrogel 1, 17.7 ± 3.9 μl for Hydrogel 2, 20.1 ± 6.4 μl for PRV and 12.5 ± 4.2 μl for the control and were considerably smaller than the intended injection volume of 30 µl. The control substance, 0.5% sodium-HA, was excluded from further longitudinal analysis due to its rapid degradation and dissipation into the surrounding tissue. In Fig. 1, the time courses of the mean absolute deposit volumes over the study period of 24 weeks are shown.

Analysis of OCT data revealed that the volumes of all deposits increased within the first two weeks (PRV) or the first four to six weeks (test hydrogels) with respect to the initially injected volume at T0. A mean increase of Hydrogel 1 and Hydrogel 2 of 10.1 ± 5.1 μl and 7.9 ± 4.7 μl, respectively, could be detected within the first weeks, while PRV increased on average 13.4 ± 6.7 μl. OCT could show a significant difference in this mean increase between PRV and Hydrogel 2 (p = 0.005), but not between PRV versus Hydrogel 1 (p = 0.131) and Hydrogel 1 versus Hydrogel 2 (p = 0.393). Analysis of the volumetric OCT data revealed the fastest absolute increase and the highest maximum volume, both relatively and absolutely, for PRV deposits. The mean maximum volumes were 75 ± 39% (Hydrogel 1), 77 ± 38% (Hydrogel 2) and 95 ± 61% (PRV) larger than the initial volumes on the first study day, with no significant difference between the different hydrogels (p = 0.273). The volumetric behavior of the hydrogels is summarized in Table 1.

**Table 1:** Volumetric behavior of the different deposits.

Filler	V increase (μl)	V max ± SD (μl)	V max_rel (%)	T max (weeks)	T 1⁄2 (weeks)
Hydrogel 1	10.1 ± 5.1	35.8 ± 4.4	175 ± 39	4.6 ± 3.8	27.2 ± 13.6
Hydrogel 2	7.9 ± 4.7	30.2 ± 5.1	177 ± 38	5.0 ± 2.9	31.5 ± 17.2
PRV	13.4 ± 6.7	36.8 ± 8.4	195 ± 61	3.3 ± 1.7	21.4 ± 12.0

All values are given as means ± SD. Based on the automatic segmentation, the following parameters were calculated: Volume (V) increase, maximum volume (V max), relative maximum volume as compared to baseline (V max_rel), time point of maximum volume (T max), half-live (T 1⁄2) calculated from an exponential fit to the data.

**Figure 2:**
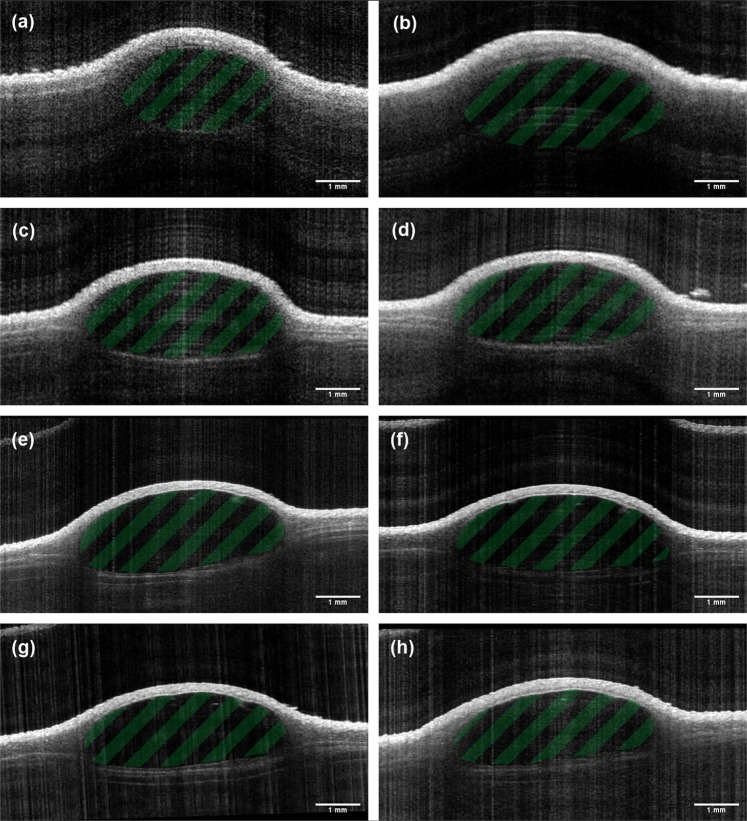
Visualization of a Hydrogel 2 soft tissue filler deposit located in the subcutis and its development over time using OCT. Data was acquired (**a**) 0, (**b**) 2, (**c**) 4, (**d**) 8, (**e**) 12, (**f**) 16, (**g**) 20 and (**h**) 24 weeks after injection. Cross sectional images 2, 4 and 8 weeks after injection reveal an increase in volume through the more nodular form of the deposit and the increased area (due to water uptake within the first weeks), as compared to the cross section obtained directly after injection. At weeks 12 to 24 (e-h), the deposit appears smaller and levels out.

The mean half-lifes T 1/2, i.e. the time from reaching the maximum volume to reduction to half of its size, as calculated from an exponential fit to the experimental data, were 27.2 ± 13.6 weeks (Hydrogel 1), 31.5 ± 17.2 weeks (Hydrogel 2) and 21.4 ± 12.0 weeks (PRV), respectively. While differences in degradation rate of the different substances in individual mice could be observed, the differences in mean half-lifes calculated from the averages of all deposits were not significant. The results of this analysis are summarized in Table 2. In Fig. 2, cross-sectional images of a Hydrogel 2 deposit are depicted and reveal the volumetric increase within the early study period and the slow degradation after the maximum volume has been reached.

**Table 2:** Differences of mean half-life T 1/2 (weeks) between the hydrogels with p-values given by ANOVA with Tukey post-hoc test.

Filler	ΔT 1⁄2 (weeks)	p-values
PRV versus Hydrogel 1	−5.7	0.395
PRV versus Hydrogel 2	−10.0	0.056
Hydrogel 1 versus Hydrogel 2	−4.3	0.589

**Figure 3:**
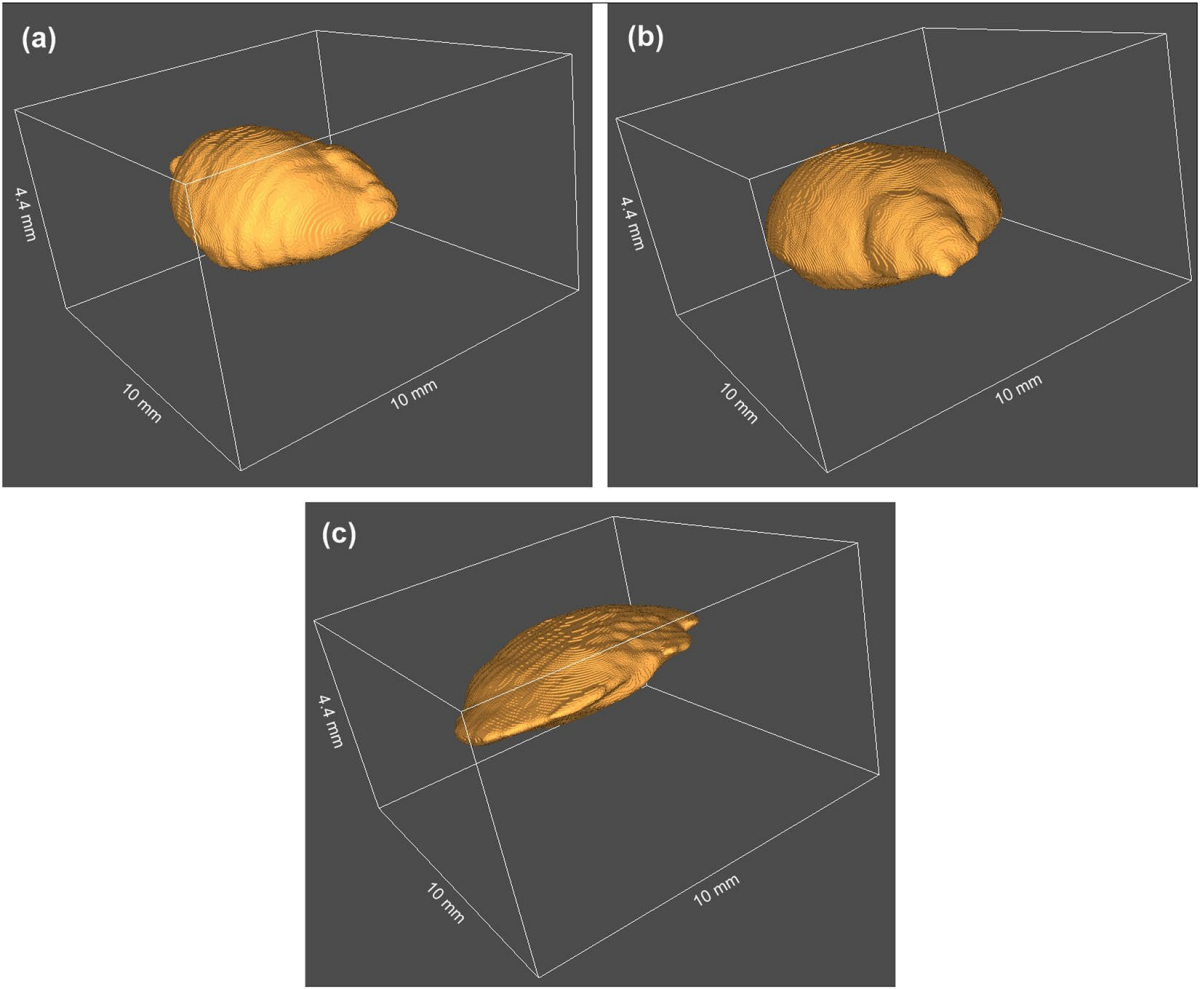
Three-dimensional representations of soft tissue filler deposits 24 weeks after injection. Graphics show (**a**) Hydrogel 1, (**b**) Hydrogel 2 and (**c**) PRV. OCT revealed a nodular shape of the test hydrogel’s deposits, caused by higher viscosity, while the lower viscosity of the PRV leads to a flat ellipsoid shape.

In Fig. 3, three-dimensional representations of the filler volumes 24 weeks after injection as obtained from the segmentation of the OCT data are shown and reveal different shapes of the fillers. Both test hydrogel deposits appear nodular in shape, whereas the PRV deposits show a more widespread, flat structure.

**Figure 4:**
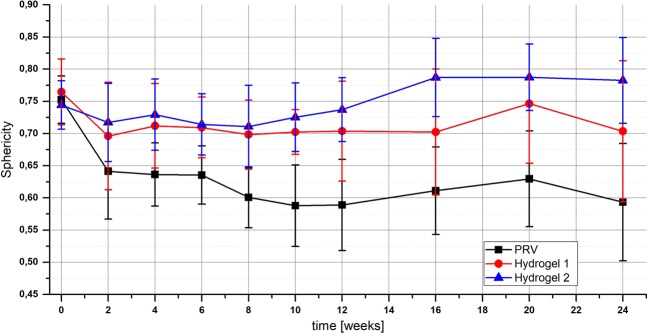
Sphericity of the different test substances (PRV, Hydrogel 1 and Hydrogel 2) over the study period of 24 weeks as calculated based on the three-dimensional OCT data. Error bars represent the standard deviation.

Analysis of the sphericity S, calculated based on the three-dimensional OCT data, revealed differences in shape and its changes over time (Fig. 4). On the injection day (T0), no significant difference between substances was found (S_mean_ = 0.75 ± 0.04). From study week two (T2) onwards, deposits of both test hydrogels revealed a significantly different, larger sphericity than PRV deposits (T2: PRV vs. Hydrogel 1 p = 0.049; PRV vs. Hydrogel 2 p = 0.003; T4 onwards: p < 0.001 for both pairs). Sixteen and 24 weeks after injection, the sphericity of Hydrogel 1 also differed significantly from Hydrogel 2 (T16: p = 0.001; T24: p = 0.012).

Comparing injection sites, filler deposits at the two caudal positions tend to have a higher sphericity than those at the two cranial positions, with differences slightly increasing over time but not reaching statistical significance. For the final measurement 24 weeks after injection, the differences for the two dorsal locations did not reach statistical significance for Hydrogel 2 (cranial S = 0.77 ± 0.05, caudal S = 0.80 ± 0.08, p = 0.296), but were significant for PRV (cranial S = 0.55 ± 0.10, caudal S = 0.64 ± 0.06, p = 0.010) and Hydrogel 1 (cranial S = 0.65 ± 0.12, caudal S = 0.76 ± 0.05, p = 0.013).

**Figure 5:**
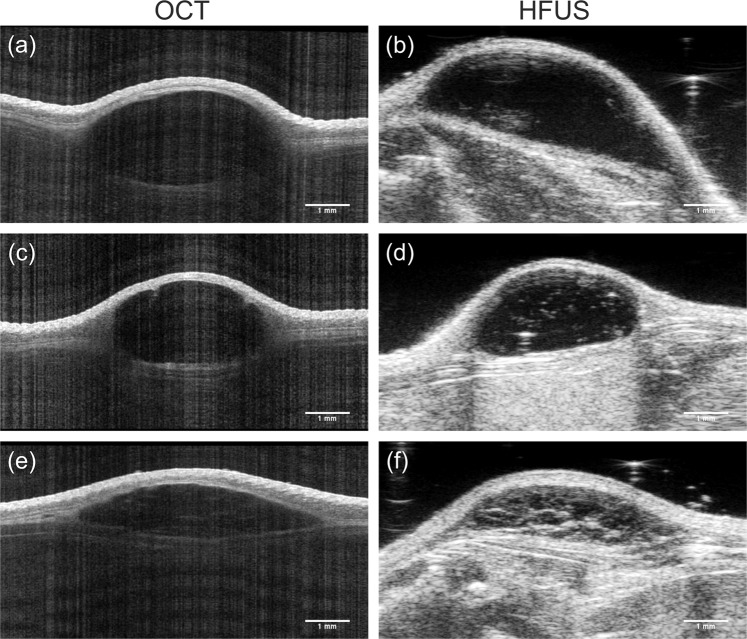
*In vivo* soft tissue filler imaging 24 weeks after injection, recorded via OCT and HFUS. Cross-sectional images of (**a,b**) Hydrogel 1, (**c,d**) Hydrogel 2 and (**e,f**) PRV deposits reveal similar visualization of the deposit shape by both imaging modalities.

Exemplary cross-sectional images of the three different materials yielded from OCT and HFUS measurements are depicted in Fig. 5. In OCT, the injected hydrogels are low scattering, thus, appearing almost homogeneously black with only minor internal reflectors, while stronger internal echoes in ultrasound imaging lead to a heterogeneous appearance of the volume.

**Figure 6:**
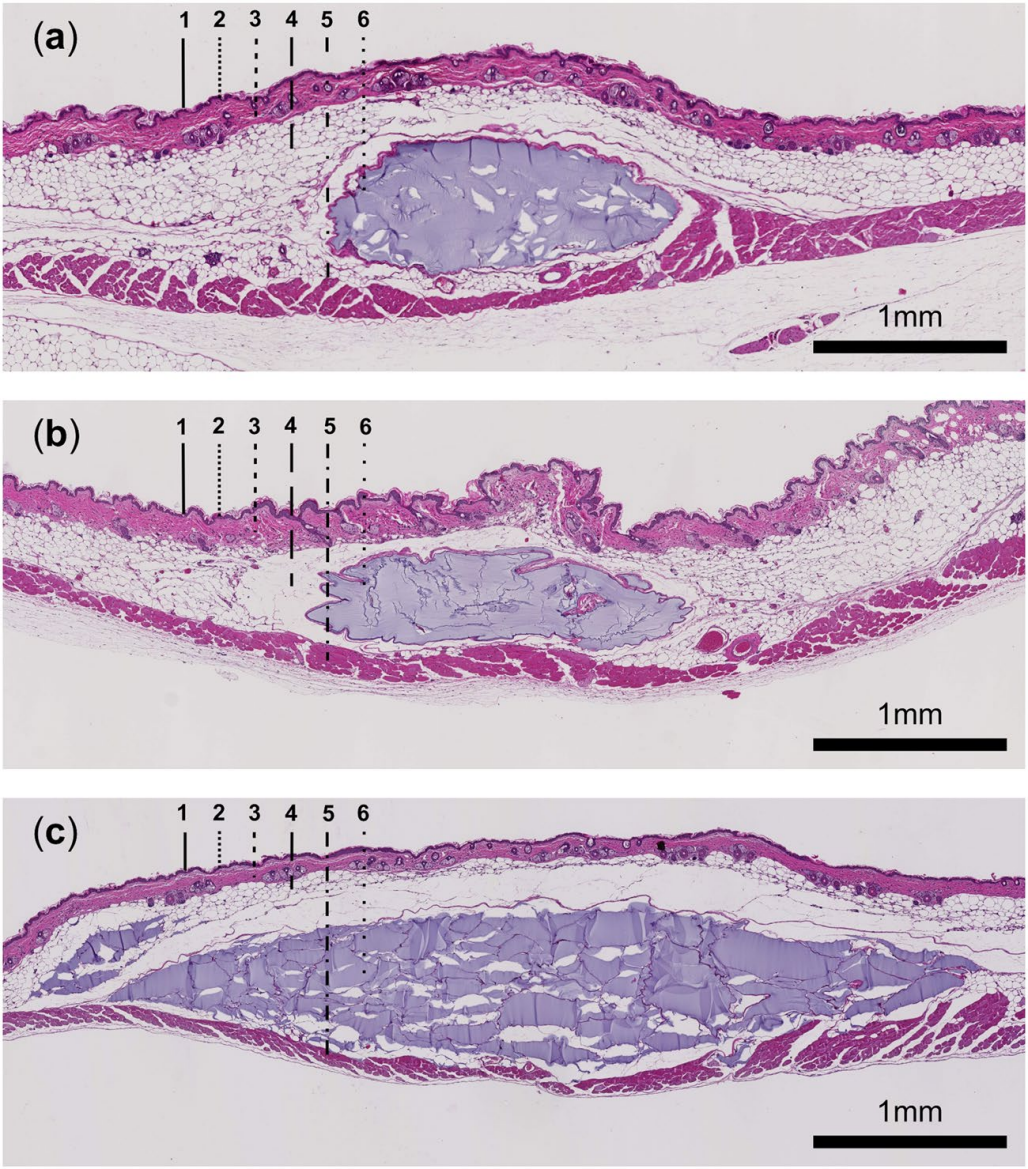
Representative H&E staining of (**a**) Hydrogel 1, (**b**) Hydrogel 2 and (**c**) PRV deposits in murine skin at week 24 (Magnification: 40x). All depicted deposits are located in the subcutis. Dashed black lines in images indicate the following skin layers and structures: (1) epidermis, (2) papillary dermis, (3) reticular dermis, (4) subcutis, (5) panniculus carnosus and (6) hydrogel deposit.

**Figure 7:**
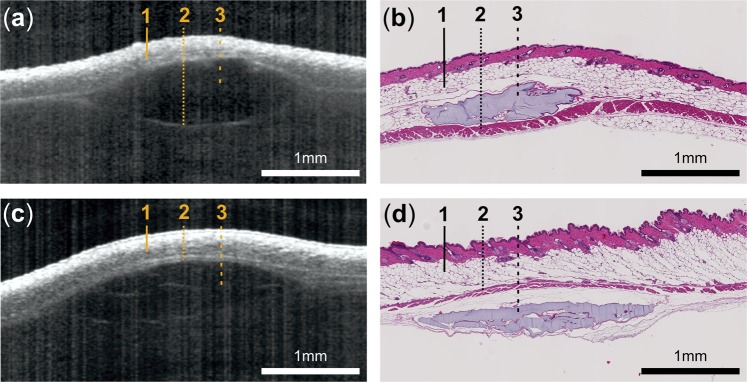
*In vivo* OCT imaging revealing the exact position of the deposit within the skin (**a**) subcutaneous and (**c**) submuscular. Histological images obtained at the last study day confirm the location of the filler in (**b**) the subcutis and (**d**) below the panniculus carnosus. Dashed lines in images indicate the following skin layers and structures: (1) subcutis, (2) panniculus carnosus and (3) hydrogel deposit.

The histological specimens were obtained on the last study day in week 24. In Fig. 6, exemplary hematoxylin and eosin (H&E) stainings of murine skin containing the intradermal deposits are depicted, visualizing the distinct shapes of the different hydrogels as detected with OCT and HFUS. Furthermore, histologic data confirmed the position of the deposits in the dorsal subcutis of all mice, except one, in which the applied hydrogel was located below the panniculus carnosus (Fig. 7).

## Discussion

The current study demonstrated the applicability of a custom-built dermatologic OCT system for obtaining qualitative and quantitative data on intradermal volumes and their geometries in the murine skin *in vivo*. The acquired data in combination with an automatic approach for segmentation of the structures revealed differences in the volumetric behavior of different test substances at different time points over a study period of six months.

Major factors determining the volumetric behavior of the test hydrogels are their polymer content, viscosity and degree of cross-linking. The higher HA content of PRV results in an increased water binding capacity, explaining the faster and stronger (PRV: 13.4 ± 6.7 μl vs. Hydrogel 1: 10.1 ± 5.1 μl and Hydrogel 2: 7.9 ± 4.7 μl) volumetric increase within the first weeks, also resulting in the highest maximum volume. In general, OCT revealed more stability of the deposits of the test hydrogels 1 and 2 over time. However, after reaching the peak volume, all deposits were steadily degraded. While in some individual mice distinct hydrogels revealed different degradation rates, the average degradation rates and half-lifes, calculated from the data set of the whole study population, were not significantly different. This finding indicates that the longevity and degradation of soft tissue fillers is highly individual and can depend on many factors, including the shape and the position of the deposit within the skin. Herein lies the major advantage of non-invasive imaging approaches like OCT, allowing to repeatedly study an individual subject over long time periods.

In the current study, volumetric OCT data revealed characteristic geometries of the different hydrogel deposits that can be attributed to differences in their viscoelastic nature. PRV deposits presented themselves in a flat ellipsoidal shape, whereas the test hydrogels appeared nodular in shape. The different ratios between surface and volume caused by these geometries might also be the reason for the observed variations in degradation. Since none of the deposits revealed geometries of higher complexity with surface irregularities or protuberances, the sphericity, which is a quantitative measure for the similarity of a filler with a perfect sphere, could provide an insight into the test substances’ distribution and compactness under the skin and allowed for their differentiation. While on the injection day the sphericities were similar, from the second week onwards, differences in viscosity influencing both the distribution and degradation of the filler were also reflected in significantly different sphericity results. The high precision of OCT measurements even revealed differences in geometry depending on the anatomical site of the deposit.

In addition to the assessment of the volume and shape, OCT measurements revealed the exact position of the injected materials. While the majority of the fillers were placed in the subcutis, in one mouse three-dimensional OCT data visualized a sub-muscular deposit. The higher reflectivity of the panniculus carnosus allowed easy localization of the deposit above or below the muscle. The localization of the deposits within the different skin layers could be confirmed by the HFUS measurements as well as histologic analysis, both performed on the last study day. When assessing the boundaries of the deposits, OCT and HFUS data revealed characteristic differences. Even though OCT cross-sectional data in general, due to the non-contact nature of the technology and the low optical powers employed for imaging, showed lower contrast and signal-to-noise-ratios, the boundary between the injected material and the surrounding tissue was visualized very distinctly.

The difference between HFUS and OCT image data can be explained, firstly, by the superior resolution of OCT in comparison to HFUS (axial resolution: OCT 6.5 µm vs. HFUS 30 µm). Furthermore, differences in the technical principles and different contrast mechanisms might contribute to this finding. The contrast in HFUS is based on variations in the acoustic impedances of tissues and materials, respectively, and thus on mechanical properties. OCT, on the other hand, relies on the optical properties and detects changes in the refractive index within the probed tissue. Due to the high water content of both the filler material as well as the surrounding tissue, the change in mechanical properties appears to be smoother, leading to a less distinct boundary, particularly at the superficial anterior side of the deposit. In OCT, on the other hand, the precise localization of the refractive index change at the border between skin tissue and filler material provides a clear visualization of the deposit boundary. These differences in reflectance pattern and optical properties are not only found, as in the current case, for skin tissue and hydrogels but also in various cell types. Boone and co-workers have shown that the analysis of optical properties such as the relative attenuation factor of different skin layers normalized to the entrance signal, allowed for differentiation of melanocytic skin lesions^29^. Applying an optical properties extraction algorithm that is computing individual optical attributes like tissue-scattering coefficient and absorption coefficient from the acquired OCT signal, Turani and co-workers reported a sensitivity of 97% and specificity of 98% for differentiation of melanoma from benign nevi^30^. The combination of the high-resolution morphological imaging provided by OCT, together with its high sensitivity to variations in optical properties indicates the technology’s high potential for dermatologic diagnostics.

Hydrogels, in the present study utilized as test substances, are primarily employed for soft tissue augmentation to reduce the signs of aging^31^, but also to improve the quality of life in patients with chronic diseases^32,33^ or after surgery^34^. However, recently the application of hydrogels as drug delivery systems, especially for targeted therapies of cancer or osteoarthritis, has been investigated. Here, the hydrogels facilitate a controlled and targeted drug release^35,36^. Besides other factors, such as the drug loading capacities, quantitative parameters as assessed in the current study might determine whether a filler qualifies as a drug delivery system. In future studies, the longitudinal measurement of the deposit’s volume in combination with conventional pharmacokinetic measurements would allow for the obtaining of a complete drug-release profile.

One limitation needs to be considered when interpreting the results of our study and their clinical implications. The main field of application of dermal OCT is the imaging of skin cancer. Here, melanocytic skin lesions can present characteristic features depending on whether it is non-melanoma^37^ or melanoma^38,39^ skin cancer. Further, the presence or absence of vessels and the pigmentation of the skin play an important role for the appearance of the lesion in OCT tomograms. The injected hydrogel deposits, on the other hand, revealed a homogeneous structure with predominantly ellipsoidal geometry, absence of surface irregularities and low internal scattering. Thus, their geometry and scattering properties might not be comparable to those of lesions in the skin. While the precision for their volumetric assessment remains unaffected hereof, additional parameters to the sphericity were to be employed for characterization of their shape. Further, if an automatic segmentation based on a neural network as employed in the current study is desired, training datasets representing the variability of different skin lesions are needed.

In conclusion, dermatologic OCT provided both qualitative and quantitative data about changes in volume and geometry of intradermal deposits in a mouse model. The acquired three-dimensional data revealed differences in shape and longitudinal behavior of different hydrogels. Beside its usefulness for treatment planning and evaluation in soft tissue augmentation, such data could be beneficial for assessing the pharmacokinetics of drug-delivering hydrogels by their volumetric assessment. In addition, by providing high-resolution morphological data and quantitative assessment of dermal structures with high accuracy and precision, OCT can support dermatologic diagnostics and complement the standard histologic examination.

## Materials and Methods

### Experimental design

OCT measurements of three soft tissue fillers and a control substance (sodium hyaluronate, 0.5%) were performed over a time period of six months in a randomized, masked and placebo-controlled study design, followed by high-frequency ultrasound imaging and histologic analysis. Four filler deposits were injected at four positions at the dorsum of each mouse: A – left cranial, B – right cranial, C – left caudal, D – right caudal. OCT measurements were performed at baseline before filler injection, immediately after injection (T0) and at time points 2, 4, 6, 8, 10, 12, 16, 20 and 24 weeks after injection (T2 - T24) in order to assess volumetric changes and longevity.

### Animals

In total, 22 female BALB/c mice, four to eight months of age with a mean initial body weight of 19 ± 1 g were obtained from Janvier Labs (Janvier Labs, Le Genest-Saint-Isle, France). The animals were kept under controlled, standardized conditions (artificial light/dark cycle 12:12, room temperature 22 ± 2 °C, humidity 55 ± 10%) in groups of four at the Center for Biomedical Research, Medical University Vienna, Austria. Mice were fed with a commercial pelleted diet (ssniff K-H, Soest, Germany), and tap water was supplied *ad libitum*. All animal procedures of this study followed the ARRIVE guidelines and the EU Directive 2010/63/EU for animal experiments^40^. All procedures were approved by the local Animal Welfare Committee and the Austrian Federal Ministry of Science, Research and Economy (GZ66.009/0380-WF/V/3b/2016, BMWFW-66.009/0299-WF/V/3b/2017).

### Injection of hyaluronic acid soft-tissue fillers

Two test hydrogels (Hydrogel 1 and 2), a commercially available HA filler (PRV, Princess VOLUME, Croma Pharma, Leobendorf, Austria) and 0.5% sodium-HA serving as control were investigated in this study. Fillers differed in their HA content, degree and method of cross-linking and their viscoelastic nature. An intended volume of 30 µl was injected into the dermis using a 250 µl syringe (Hamilton, Reno, Nevada, USA) and a 27 gauge needle. Each mouse received one deposit of each filler. The injection site of each filler type was randomized. Both filler injection and data evaluation were operator blinded.

Before each measurement, dorsal fur was shaved using an Aesculap GT420 Isis shaver (B. Braun Vet Care GmbH, Germany) and remaining fur was removed with a chemical depilatory (Veet crème sensitive, Reckitt Benckiser, Slough, UK). The injection of the fillers was performed aseptically under general anesthesia using intraperitoneal injection of ketamine (80–100 mg/kg) and xylazine (10 mg/kg). Additionally, metamizol (100 mg/kg) was administered for analgesia. For repeated imaging, anesthesia was administered through a nose cone (Isofluran, 2% induction and 1.5% maintenance).

### Imaging modalities

#### Optical coherence tomography

Optical coherence tomography images were recorded using a custom-built swept-source OCT system based on an akinetic light source (Insight Photonic Solutions, Inc., Lafayette, Colorado, USA) with a flat spectrum centered at 1310 nm and a bandwidth of 87 nm. Experimental setup of the OCT system and data processing have been published previously^41^. The imaging system provided resolutions of 9 µm and 22 µm in the axial and lateral direction, respectively. For assessment of intradermal filler volume, three-dimensional data sets over a skin area of 10 mm × 10 mm, each comprising 512 × 1500 × 1536 voxels, were recorded. The acquisition time for one volumetric data set was 14 seconds. The incident power of the probe beam onto the tissue was set to 33 mW, which is well within the safety limit recommended by the International Electrotechnical Commission^42^.

### High-frequency ultrasound

High-frequency ultrasound (Vevo2100, FUJIFILM VisualSonics Inc., Toronto, Canada) was used for visualizing position within the skin and shape of the intradermal volumes at week 24, using a high-frequency transducer probe (VisualSonics MS700, FUJIFILM VisualSonics, Inc., Toronto, Canada) with a frequency range of 30–70 MHz, which yielded a characteristic resolution of 30 µm and a maximum image depth of 9.7 mm^43^.

### Data analysis

Dermal filler volumes were determined from OCT volume data sets by automatic segmentation of the filler area within the cross-sectional images and accounting for the scan range in both lateral dimensions. A machine-learning based algorithm for segmenting the deposits has been developed in order to give a three-dimensional representation of the volumes^41^. All automatic filler segmentations were visually examined by an experienced operator and manually corrected in those cases where the automatic algorithm failed to correctly capture the filler area.

For the evaluation of the shape of the deposit, the sphericity S, defined as the ratio between the surface area A of a sphere with the same volume V as the given deposit and the surface area of the actual deposit, was calculated^44^:

1 $${\rm{S}}=\frac{{\pi }^{1/3}{(6\cdot {\rm{V}})}^{2/3}}{{\rm{A}}}$$

The surface area A was calculated as the sum of all voxels at the boundary of the deposit weighted depending on the configuration of individual voxel surfaces facing the boundary^45,46^. According to Eq. (1), a spherical deposit would have a sphericity of 1, while any deviation from that shape leads to a value smaller than 1.

### Histologic evaluation

Histological analyses of the soft tissue filler deposits in the murine skin were performed on the last study day in week 24. The skin samples containing the deposit, including epidermis and dermis, were excised and fixed in 4% formalin (Roti-Histofix 4%, Carl Roth GmbH + Co. KG, Karlsruhe, Germany) for 24 hours, dehydrated in an ascending ethanol series and subsequently embedded in paraffin. The histological sections were stained with hematoxylin and eosin (H&E) at the University of Veterinary Medicine, Vienna, Austria. A Hamamatsu NanoZoomer 2.0 HT slide scanner (Hamamatsu Photonics Deutschland GmbH, Ammersee, Germany) was used to scan the H&E slides using the Hamamatsu NDP.view2 software. The scans (10–40x magnification) were exported as jpeg-images via the NDPI tool plugin of the Fiji program (National Institutes of Health, available in the public domain at https://rsbweb.nih.gov/ij/). The histological analyses were conducted via light microscopy and Fiji4 based on the digital H&E slides.

### Statistical analysis

All statistical analyses were performed in SPSS (IBM SPSS Statistics, Version 25). The volume increase rate due to water uptake within the first weeks after injection was calculated by correlating injection volume and maximum volume after injection. The half-life was calculated from an exponential fit starting at the maximum volume.

One-way analysis of variance (ANOVA) was used to test for differences in relative volume, time at which maximum volume was reached, half-life and increase rate and sphericity among multiple filler groups. A post-hoc analysis was performed (Tukey’s honestly significant difference test) to correct for multiple testing and identification of differences between specific groups. Statistical significance was accepted at p < 0.05.

## Data Availability

Data generated in the present study are available from the corresponding author upon reasonable request.
